# The Health and Well-being Impacts of Community Shared Meal Programs for Older Populations: A Scoping Review

**DOI:** 10.1093/geroni/igac068

**Published:** 2022-10-27

**Authors:** Georgia Middleton, Karen A Patterson, Eimear Muir-Cochrane, Stefania Velardo, Fidelma McCorry, John Coveney

**Affiliations:** Caring Futures Institute, College of Nursing and Health Sciences, Flinders University, Adelaide, South Australia, Australia; College of Nursing and Health Sciences, Flinders University, Adelaide, South Australia, Australia; College of Nursing and Health Sciences, Flinders University, Adelaide, South Australia, Australia; College of Education, Psychology and Social Work, Flinders University, Adelaide, South Australia, Australia; Centre of Research Excellence in Translating Nutritional Science to Good Health, The University of Adelaide, Adelaide, South Australia, Australia; Caring Futures Institute, College of Nursing and Health Sciences, Flinders University, Adelaide, South Australia, Australia

**Keywords:** Mental health, Shared meals, Social eating, Review

## Abstract

**Background and Objectives:**

There are social and economic benefits to supporting individuals to live independently for as long as possible. Structured shared meal programs provide opportunities for older individuals to connect in their communities and likely impact their health and well-being. Research in this area has not been summarized in recent years. This scoping review was undertaken to explore the impact shared meal programs may have for older community-dwelling adults.

**Research Design and Methods:**

Nine databases were systematically searched in 2020, and 5,996 unique studies were identified. Two independent reviewers screened titles, abstracts, and full text for inclusion. Reference lists of included papers were hand searched, and the search was updated in 2021. Eighteen studies were included in the final review.

**Results:**

Studies were published between 1980 and 2021 and most were published in the United States. Most studies were cross-sectional, two adopted a qualitative design, one a cohort design. Significant associations were reported between shared meal programs and improved dietary intake; however, minimal improvements were reported for physical health measures. The programs had a positive impact on attendees’ social networks and perceived well-being.

**Discussion and Implications:**

Structured shared meal programs show promise in supporting the health and well-being of older adults in the community. They provide additional nutrition, opportunities for social connection, and are perceived to contribute to perceived well-being. More investigation is required to understand how these programs work to facilitate health and well-being, and how they can best be used to improve health outcomes for older populations.

**Translational Significance:** The potential impact of shared meal programs on the health and well-being of older adults is not yet known. This review aimed to identify whether these programs could be a potential approach for promoting health and well-being in older adults. The findings identified that these programs may have this potential; however, more work is required to understand the value of these programs beyond the nutritional value of the food provided. Once we have this understanding, we can improve current programs and develop future programs that will enhance the health, well-being, and life satisfaction of older adults.

## Background and Objectives

In 2019, 703 million individuals across the globe were aged 65 years or over (United Nations, 2019). This is projected to grow to 1.5 billion in 2050 due to the increases in life expectancy seen across the world (United Nations, 2019; WHO 2011). Aging is commonly associated with deteriorations in health and mobility, and increased frailty and disability (Ferrucci et al. 2016; Steed et al. 2007), in some instances demanding higher levels of care. Not surprisingly, there are clear social and economic benefits associated with keeping individuals in their own homes and living independently for as long as possible (WHO 2011). However, with declining support from families, there is an increased need for community-based services and systems to support independent living and “aging in place” (Bigonnesse & Chaudhury, 2020).

Inadequate nutrition is a key risk factor contributing to development and worsening of chronic conditions in older adults (Keller, 2004; MacIntosh et al., 2000). Good nutrition reduces the risk of malnutrition and diet-related illnesses, maintains muscle mass and cognitive performance, and prevents frailty among older adults (Boulos et al., 2017; Drewnowski & Shultz, 2001; Klimova et al., 2020; Payette & Shatenstein, 2005). The mechanisms contributing to inadequate nutrition in older adults are complex, and reduced dietary intake is a known key contributor (Ahmed & Haboubi, 2010). Reduced dietary intake is often attributed to the physiological changes of aging, along with reductions in sensitivity of tastebuds, appetite, and desire to eat (Ahmed & Haboubi, 2010; Donini et al., 2003; Whitelock & Ensaff, 2018). However, other factors that contribute to reduced dietary intake include physical limitations, loss of a spouse, loneliness, and social isolation (Andersen & Brunner, 2020; Bloom et al., 2017; Donini et al., 2003; Whitelock & Ensaff, 2018). These factors can encourage meal skipping, preparation of simple meals, avoidance of certain foods, and reliance on ready meals (Whitelock & Ensaff, 2018).

In addition to potentially comprising dietary intake, loneliness and social isolation can negatively impact the health and well-being of older individuals (Goll et al., 2015; Luanaigh & Lawlor, 2008; Yang et al., 2016). Both social isolation and loneliness are linked to a range of negative health outcomes, such as poor psychological well-being (including increased levels of depression and anxiety), increased mortality rates, and cognitive decline (Goll et al., 2015; Luanaigh & Lawlor, 2008; Nicholson, 2012; Yang et al., 2016). Global estimates of older adults experiencing loneliness and social isolation are not known; however, it is estimated to be high, ranging from 10% to 43% (Nicholson, 2012; WHO, 2021). Common life changes associated with aging, such as retirement, loss of a spouse or loved one, the passing of friends and neighbors, and adult children moving away, can all contribute to feelings of social isolation and loneliness (Goll et al., 2015; Grenade & Boldy, 2008; Nicholson, 2012). This has been exacerbated by the COVID-19 pandemic and its associated imposed isolation restrictions and physical distancing measures (Hwang et al., 2020; WHO, 2021).

Commensality, or the sharing of food in a social environment, has been shown to provide benefits for both social and physical health (Dunbar, 2017; Jönsson et al., 2021; Ochs & Shohet, 2006). Commensal eating occasions are noteworthy, given they could concurrently combat both inadequate nutrition and experiences of loneliness and social isolation in older adults. Social facilitation of eating, whereby individuals eat more food in the presence of familiar others, has been demonstrated in prior research (Ruddock et al., 2019), with increases in food intake up to 60% specifically in older populations (McAlpine et al., 2003). There is also evidence that sharing food enhances the strength of social connections and bonds (Dunbar, 2017). Sharing meals with others symbolizes community, provides opportunities for social interactions, information exchange, and supportive relationships (Andersen & Brunner, 2020; Kushida et al., 2020). Research has shown that those who engage regularly in commensal eating events feel happier, have higher life satisfaction, are more engaged, and have more friends they can depend on (Dunbar, 2017).

Structured, shared meal occasions in the community offer an opportunity for older individuals to address and mediate the risk factors associated with poor nutrition, social isolation, and loneliness (Herne 2009; Stehouwer 2014). Shared meal programs for older individuals have existed in the community, either formally or informally, for many years, in various forms. These programs are often subsidized, offered to older individuals to provide nutrition through a shared meal, and in some cases also include opportunities for physical activity, information, and other supports (Lloyd & Wellman, 2015). Shared meal programs have been reported to foster social connections and interactions, provide companionship, offer support, and contribute to feelings of a better quality of life (Herne, 2009; Kirk et al., 2001; Middleton et al. 2022; Stehouwer, 2014). However, a formal review of the impact these types of occasions have on the health and well-being of older individuals living in the community has not yet been undertaken.

Previous literature reviews have explored aspects of shared meal programs (Beck et al., 2020; Herne, 2009; Stehouwer, 2014); however, to our knowledge, there has not been a review exploring the impact of structured shared meal programs on the health and well-being of older adults in the community. In Stehouwer et al.’s review, they noted that the majority of research in this space focuses on nutritional and physical outcomes and highlighted a gap in the literature on the psychosocial outcomes of participating in such programs (Stehouwer, 2014). Therefore, this review set out to explore the potential impact structured shared meal programs may have on older adults living in the community, including outcomes beyond just nutrition and physical health, including a specific focus on psychosocial health.

## Research Question, Aims, and Objectives

This review set out to answer the following question: What is known from the existing literature about the impact structured commensal eating events have on adults over 60 years of age in the community?

The objectives of this scoping review are as follows:

1. To identify the scope of relevant literature in this field
2. To explore the impact structured commensal eating events have on older individuals in the community
3. To explore the health or well-being outcomes they may gain from attending such events

## Research Design and Methods

Scoping reviews are appropriate when an area of research has not yet been extensively reviewed, and when looking to identify gaps in existing literature (Arksey & O’Malley, 2005). As such, it was deemed that a scoping review would be the most suitable approach to address our research question and objectives. Arskey and O’Malley’s scoping review framework, Levac and colleagues’ expanded version (Arksey & O’Malley, 2005; Levac et al., 2010), and Tricco and colleagues’ PRISMA extension for Scoping Reviews (PRISMA-ScR) were used to guide and structure this review (Arksey & O’Malley, 2005; Levac et al., 2010; Tricco et al., 2018). This manuscript follows the reporting guidelines of the PRISMA-ScR Checklist (Tricco et al., 2018).

### Study Eligibility

#### Population

To be included in this review, the average age of study populations had to be ≥60 years, and participants had to be living in the community. This review excluded studies where the average age of participants was less than 60 years, those living in aged or residential care facilities, or residing in hospital.

#### Intervention

The intervention of interest was structured shared meal programs with peers, not with family, volunteers, health professionals, or similar. Shared meals had to be structured events held regularly in the community, but not in residential care or hospital facilities. Studies focused exclusively on shopping, cooking, or home-delivered programs were excluded, unless they included or compared against a shared meal program component. Studies evaluating interventions held at shared meal programs were not included if they did not measure the outcomes of the shared meal program itself.

#### Control

It was not a criterion that all included studies contain an intervention and control study sample. For studies that did, the control group was those who did not attend the shared meal program, or those who participated in other meal services that did not contain a social component.

#### Outcome

Studies had to report outcomes related to the impact of the shared meal programs on objective or subjective measures of health or well-being. This review was not interested in evaluations of the shared meal programs themselves (e.g., the quality of the food, the quality of the service), but rather the impact the shared meal programs had on participants (e.g., changes to dietary intake or social contact). Studies exclusively reporting descriptive characteristics of participants attending shared meal programs were excluded.

#### Study design

This review considered most study designs. Excluded were systematic reviews, meta-analyses, and umbrella reviews. Non-original articles were excluded, such as book chapters, editorials, case studies, conference proceedings, and abstracts.

### Information Sources

The databases searched include Medline (via OVID SP), EmCare (via OVID SP), CINAHL, Scopus, Web of Science Core Collection (via ISI Web of Science), ProQuest (social sciences and Health & Medicine collections), and Informit. Unpublished and gray literature studies were located through Google advanced and GreyLit databases.

### Search Strategy

An initial limited search of Medline and EmCare was used to develop the search strategy and identify key search terms. Key search terms were combined using the AND/OR operators for the population (elder*, geriatric*, gerontol*, old age*, grandparent*, retire*, pensioner*, senior*, old*, age*, aging, person, people*, adult*, resident* m?n, wom?n, male*, female*) and setting (social, group, structure*, formal, commensal, communit*, communal, congregate*, shar*, meal*, food*, eating, dining), and were limited to English. The search strategy was run in the databases listed above, adjusted as needed, on June 5, 2020 and updated on June 11, 2021. A full electronic search strategy for Ovid Medline is provided in Supplementary Table 1. The reference lists of included papers were screened to identify any additional studies and search alerts were set up in several databases to ensure relevant papers were captured.

### Study Selection

**Figure 1.**
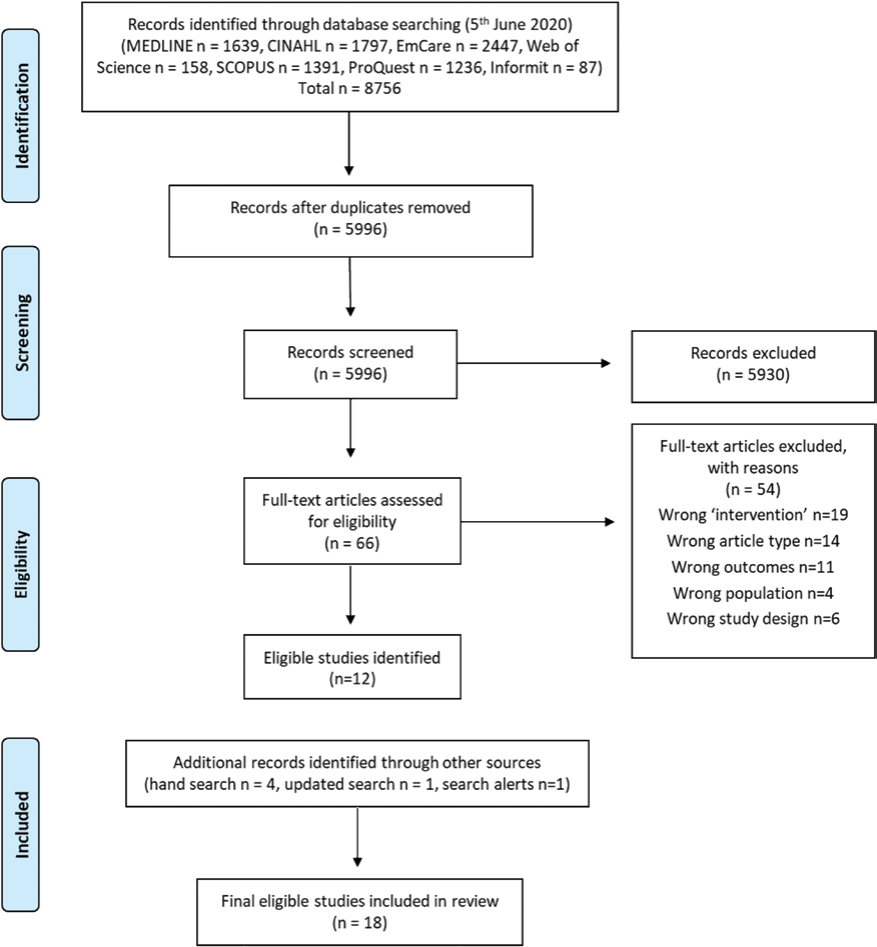
PRISMA flowchart of search strategy and included papers.

After running the searches in the selected databases, all identified citations were uploaded into EndNote (Clarivate Analytics, 2022) before being exported to Covidence systematic review software (Veritas Health Innovation, 2022). Duplicates were removed, and title and abstract screening were undertaken against predetermined inclusion and exclusion criteria by two independent reviewers, with conflicts resolved by a third reviewer. Studies identified as relevant were read in full against predetermined inclusion and exclusion criteria by two independent reviewers and conflicts were resolved by a third reviewer. Studies excluded after full-text review were recorded, with reasons reported in the PRISMA diagram (Figure 1; Moher et al., 2009).

### Data Extraction

Data related to the scoping review objectives were extracted from included studies by one reviewer (G. Middleton) using a predetermined data-extraction spreadsheet. Extraction was conducted twice on all studies to ensure all relevant data had been captured. Data were extracted on the study population, context and setting, geographical location, methodology and methods, shared meal program, and relevant findings.

### Data Analysis and Synthesis of Studies

All data pertaining to any “impact” the shared meal programs had on participants were extracted. After data were extracted from each individual study, findings that reported similar outcomes were grouped together into categories. As this is a scoping review, no further synthesis, meta-analysis, or meta-aggregation was undertaken (Arksey & O’Malley, 2005). The synthesis findings were informed by a sequential explanatory approach, whereby the quantitative findings were extracted, synthesized, and compared, prior to the extraction, synthesis, and comparison of the qualitative findings (Pluye & Hong, 2014).

The development of the categories was iterative and underwent many changes, before being finalized as: Dietary intake/meal patterns; Nutrition status; Physical health; Social support/network; and Well-being/Quality of life. “Dietary intake/meal patterns” included outcomes related to intake or perception of intake of meal patterns and dietary or meal composition. “Nutrition status” included outcomes related to nutrition status acquired from biochemical assessment, or perceptions or assessments of nutrition risk. “Physical health” included outcomes related to any measures or perceptions of physical health or ability, such as markers of physical health (e.g., blood pressure), physical activity or ability. “Social support/network” included outcomes related to perceived levels of social support or connectedness. “Well-being/Quality of life” included outcomes of “well-being” defined as the combination of positive feelings and effective functioning in daily life (Huppert, 2009), and of “quality of life” defined as any measure of an individual’s perception of their position in life (The WHOQOL Group, 1998). The results are presented in narrative form supplemented with tables. As both the qualitative and quantitative data extracted from the included studies complemented one another, and easily fit under the five developed categories, they are presented together.

## Results

### Study Inclusion

After the processes of title, abstract, and full-text screening were undertaken, there were 12 relevant papers included in this review (Figure 1). An additional four articles were located through hand-searching of reference lists, one additional article was located through the updated search in 2021, and another was located through search alerts in 2022. Eighteen articles in total were included in this review.

### Characteristics of Included Studies

The characteristics and results of the included papers are presented in detail in Table 1 and Supplementary Table 2. The papers included in this review were published between 1980 and 2021. Two of the included studies had a qualitative design (Sheppard et al., 2018; Thomas & Emond, 2017), one had a cohort design (Keller, 2006), and the remaining fifteen had a cross-sectional design. Study samples ranged from 9 to 1,072, participant ages ranged from 51 to 103 years of age, and females made up a large proportion of participants in each study (50% to 87.5% female participants). The main sources of data collection were interview-administered surveys (Administration for Community Living [ACL], 2004, 2008, 2009, 2011–2019; Beasley et al., 2018; Choi et al., 2021; Dichiera et al., 2002; Heuberger & Wong, 2014; Huffman et al., 2017; Keller, 2006; Kohrs et al., 1980; Neyman et al., 1998; Vailas et al., 1998; Van Zandt & Fox, 1986; Ye et al., 2017), self-administered surveys (Neyman et al., 1996; Porter et al., 2016; Schultz et al., 2021; Tsofliou et al., 2020), interviews (Thomas & Emond, 2017), and focus groups (Sheppard et al., 2018). Survey tools varied depending on the outcomes measured, and other data collection tools such as anthropometric measurements, biochemical measurements, and food intake measurements were varied across studies.

**Table 1.** Study Characteristics of Included Papers

Study; country	Recruitment	Participant	Study design	Shared meal program	Tools used for data collection
Kohrs et al., 1980; United States	All persons eating at one of the five selected meal sites 2+ times per week invited to attend, random sample drawn from participants who had eaten at the site <2 times per week	*N* = 547 (59–99 y/o, 69% female)	Cross-sectional	OAA Title III CMS	Interview-administered survey (1-day food record, 24 hr recall, food frequency questionnaire), medical history, clinical examination, blood and hair samples, anthropometry
Van Zandt & Fox, 1986; United States	Subjects present on days researcher visited meal sites were recruited	*N* = 170 (60–90 y/o, 65% female)	Cross-sectional	OAA Title III CMS	Interview-administered survey
Neyman et al., 1996; United States	Recruited from “senior” centers during the congregate lunch meal, and from organization meetings held at centers	*N* = 135 (60–89 y/o, 67% female) Participants, *n* = 70 Non-participants, *n* = 65	Cross-sectional	OAA Title III CMS	Self-administered survey, blood test
Neyman et al., 1998; United States	From “senior” centers and other “senior” organizations	*N* = 80 (60–93 y/o, 76% female) CMS, *n* = 40 HDM, *n* = 40	Cross-sectional	OAA Title III CMS	Interview-administered survey (24 hr dietary recall)
Vailas et al., 1998; United States	Participants recruited through meal program employees	*N* = 155 (71–86 y/o, 73% female) CMS, *n* = 108 HDM, *n* = 47	Cross-sectional	OAA Title III CMS and HDM	Interview-administered survey (range of tools), anthropometry
Dichieria et al., 2002; United States	Random sample of national participants who access the service recruited through Area Agencies on Aging (provide client lists)	*N* = 51 (51–94 y/o, 72% female)	Cross-sectional	OAA Title III CMS	Interview-administered survey
ACL, 2003, 2004, 2008, 2009, 2011–2019; United States	Random sample of national participants who access the service recruited through Area Agencies on Aging (provide client lists)	*N* = 473–1,072 (60–85+ y/o, 61%–73% female)	Cross-sectional	OAA Title III CMS	Interview-administered NSOAAP
Keller, 2006; Canada	Through agencies providing services to the older adults	*N* = 267 (70–86 y/o, 76.4% female)	Cohort	Home care services, including congregate meal programs (CMS), Meals on Wheels	Interview-administered survey (SCREEN)
Heuberger & Wong, 2014; United States	Word of mouth, advertisement, flyers, community organizations	*N* = 1,065 (60–103 y/o, 65% female)	Cross-sectional	Congregate meals as part of the Elderly Nutrition Program	Interview-administered survey
Porter et al., 2016; United States	From 12 selected congregate meal sites	*N* = 256 (66–82 y/o, 66% female)	Cross-sectional	OAA Title III CMS (heterosexual vs. lesbian, gay, bisexual, transgender individuals)	Self-administered survey
Thomas & Emond, 2017; United Kingdom	Recruitment following presentation at selected lunch clubs	*N* = 10 (60–80 y/o, 50% female)	Qualitative	Lunch clubs specifically for older people	Food diaries, interview
Huffman et al., 2017; United States	Sampling of the 2015 Tenth Annual NSOAAP	*N* = 901 (60–>75 y/o, 68% female)	Cross-sectional secondary analysis	OAA Title III CMS	Interview-administered survey (2015 Tenth Annual NSOAPP Congregate Meals)
Ye et al., 2017; China	Random sampling from older adults’ “senior” center services utilization survey 2011	*N* = 320 (65–83 y/o, 57% female)	Cross-sectional	Community diners at Shanghai’s “senior” centers	Interview administered survey (Shanghai “senior” center service utilization survey)
Beasley et al., 2018; United States	Sampling from 2015 Tenth Annual NSOAPP, recruitment via convenience sampling from two New York City “senior” centers through flyers and announcements at lunches	National survey data*, N* = 901 (60–85+ y/o, 66.2% female) Local data, *n* = 22 (60–84 y/o, 68.2% female)	Cross-sectional secondary analysis	OAA Title III CMS	Over the phone survey (2015 Tenth Annual NSOAPP, Montreal cognitive assessment), 4-day food record, RAND 36, Short Physical Performance Battery, Jamar hand-held dynamometer
Sheppard et al., 2018; Canada	Recruited from urban “senior” center	*N* = 9 (average age 72 y/o, 78% female)	Qualitative process evaluation	“Let’s Do Lunch” program	Focus groups
Tsofliou et al., 2020; United Kingdom	Convenience sampling at the five selected lunch clubs	*N* = 39 (73–90 y/o, 56.4% female, 64.1% living alone)	Cross-sectional	Lunch clubs with for those ≥ 65 y/o	Self-administered survey (24-hr dietary recall), anthropometry
Choi et al., 2021; South Korea	Secondary analysis of national survey data, participants over 65 y/o who had eaten at an IF meal, or a home-made or purchased meal (non-IF) at a social leisure services facility were sampled	*N* = 390 (65–75+ y/o, 66.3% female, 27.7% living alone) IF, *n* = 129 Non-IF, *n* = 261	Cross-sectional	Institutional Food Service meals provided at Social Leisure Services facilities in South Korea	Sixth and seventh Korea National Health and Nutrition Examination Survey
Schultz et al., 2021; United States	Convenience sample through research team announcements to those present at the time	*N* = 136 (traditional and innovation group combined) (<60–85+ y/o, 60.3% female, 56.2% living alone) Lunch program, *n* = 26 Comparison group, *n* = 21	Cross-sectional	Encore Café innovative congregate nutrition program (innovation group), compared with traditional congregate nutrition programs (traditional group), and no lunch program (comparison group)	Survey (administration unclear)

*Note*: CMS = congregate meal services; HDM = home-delivered meals; IF = institutional food service; NSOAAP = National Survey of Older Americans Act Participants; OAA = Older Americans Act; Nutrition Services Program ; SCREEN = Seniors in the Community: Risk Evaluation for Eating and Nutrition©; y/o = years old.

Of the 18 included papers, two were conducted in the United Kingdom (Thomas & Emond, 2017; Tsofliou et al., 2020), two in Canada (Keller, 2006; Sheppard et al., 2018), one each in China (Ye et al., 2017) and South Korea (Choi et al., 2021), and the remaining 12 in the United States. All but one paper from the United States assessed the Older Americans Act (OAA) Title III Nutrition Services Program (NSP). This program provides state funding to nutrition services that support older populations, including both shared meal and home-delivered meal programs (Administration for Community Living [ACL], 2021). The ACL administers annual surveys on the OAA Title III NSP; The National Survey of Older Americans Act Participants (NSOAAP). Thirteen of these surveys are publicly available on the “AGing, Independence, and Disability (AGID) Program Data Portal,” from years 2003, 2004, 2008, 2009, 2011–2019 (ACL, 2004, 2008, 2009, 2011–2019). Results from these nationally collected surveys have been combined to present one set of results, representing the range of participants and responses from 2004 onwards (the 2003 survey did not contain data relevant to the review). Other studies that have conducted their own analyses or comparison on the data relating to congregate meal services, or who have collected their own data from the OAA Title III NSP congregate meal services, were included. The remaining studies investigated various other meal programs, many not specified beyond their service to older populations in the community (Keller, 2006; Thomas & Emond, 2017; Tsofliou et al., 2020) or being conducted in “senior” centers (Choi et al., 2021; Ye et al., 2017).

For more detail on individual study characteristics, see Table 1.

### Findings of the Review

Table 2 provides a summary of the impact on outcomes related to dietary intake and meal patterns, nutrition status, physical health, social support and network, and well-being and quality of life of the included studies.

**Table 2.** Summary of Findings of Included Papers

Study	Reported impact on outcomes related to health or well-being^a^				
	Dietary intake/meal patterns	Nutrition status	Physical health	Social support/network	Well-being/quality of life
Kohrs et al., 1980	**+ve**	**+ve**	**+ve -ve**		
Van Zandt & Fox, 1986	+ve		—	+ve	+ve
Neyman et al., 1996	**+ve**	—	—	+ve	
Neyman et al., 1998	+ve				
Vailas et al., 1998		+ve			**+ve**
Dichieria et al., 2002	**+ve**			+ve	
ACL, 2003, 2004, 2008, 2009, 2011–2019	+ve		+ve	+ve	+ve
Keller, 2006		+ve			
Heuberger & Wong, 2014	—				
Porter et al., 2016	+ve			**+ve**	
Thomas & Emond, 2017				+ve	
Huffman et al., 2017	+ve				**+ve**
Ye et al., 2017				+ve	**+ve**
Beasley et al., 2018	+ve		+ve		+ve
Sheppard et al., 2018	+ve			+ve	
Tsofliou et al., 2020	**+ve**			+ve	
Choi et al., 2021	**+ve**		—		
Schultz et al., 2021	**+ve -ve**	-ve	—		**+ve**

*Notes*: — = outcome was measured, but no difference was noted; +ve = attendance has positive impact on outcome, or more positive than non-attendance; -ve = attendance had negative impact on outcome, or more negative than non-attendance; **+ve** or **-ve** indicate this finding was statistically significant.

^a^For detailed descriptions of the findings, see Supplementary Table 2.

#### Dietary intake and meal patterns

Thirteen of the included studies quantitatively measured and reported outcomes related to dietary intake and meal patterns (ACL, 2004, 2008, 2009, 2011–2019; Beasley et al., 2018; Choi et al., 2021; Dichiera et al., 2002; Heuberger & Wong, 2014; Huffman et al., 2017; Kohrs et al., 1980; Neyman et al., 1998; Neyman et al., 1996; Porter et al., 2016; Schultz et al., 2021; Tsofliou et al., 2020; Van Zandt & Fox, 1986), and one explored this aspect using qualitative methods (Sheppard et al., 2018). Eight of these studies measured outcomes of participants who attended a shared meal program, with no comparison to those who did not attend (ACL, 2004, 2008, 2009, 2011–2019; Beasley et al., 2018; Dichiera et al., 2002; Huffman et al., 2017; Porter et al., 2016; Sheppard et al., 2018; Tsofliou et al., 2020; Van Zandt & Fox, 1986). The remaining six investigated the dietary quality and meal patterns of those who attended shared meal programs, compared with those who did not attend, did not attend frequently, or attended a different program to the program of interest (Choi et al., 2021; Heuberger & Wong, 2014; Kohrs et al., 1980; Neyman et al., 1996, 1998; Schultz et al., 2021). All but four of these studies investigated the OAA Title III NSP, one of which compared the OAA Title III NSP with their own innovative program (Schultz et al., 2021), two looked at shared meal programs for those aged over 65 years in the United Kingdom (Tsofliou et al., 2020) and Canada, respectively (Sheppard et al., 2018), and the last investigated a similar government-funded service to the OAA Title III NSP at a Social Leisure Services facility in South Korea (Choi et al., 2021).

Participants across these studies reported a change in meal patterns since attending the shared meal program under investigation (Van Zandt & Fox, 1986), enjoyed the food provided at the meal (Van Zandt & Fox, 1986), felt the program helped keep food-related costs manageable (Van Zandt & Fox, 1986), and viewed the meals as nutritionally balanced and contributing to a healthier diet (ACL, 2004, 2008, 2009, 2011–2019; Beasley et al., 2018; Dichiera et al., 2002; Huffman et al., 2017; Porter et al., 2016; Van Zandt & Fox, 1986). The most important factors of attending the meal, as rated by participants in Tsofliou et al.’s UK study, were accessing a hot meal (74.4%), eating a meal outside of the home (76.9%), eating a home-style cooked meal (71.8%), and not having to cook a meal themselves (43.6%) (Tsofliou et al., 2020). For participants in Dichieria’s study, 63% reported enjoyment of the meals as a main reason for attending the program, followed by 57% reporting the low cost of the meals as a motivator for attending (Dichiera et al., 2002). Participants in Sheppard et al.’s qualitative evaluation of the Let’s Do Lunch program in Canada described the program as an opportunity to enjoy healthy, tasty, inexpensive, and convenient meals (Sheppard et al., 2018).

In terms of contribution to dietary intake, participants in Van Zandt and Fox’s cross-sectional study assumed that the food provided at the meal was sufficient for the day, or that it would be in addition to their intake at home (Van Zandt & Fox, 1986). Huffman et al. reported some participants received >50% of their daily calories coming from the shared meal program (Huffman et al., 2017), indicating that for some participants, shared meal programs provided a significant contribution to their dietary intake. This was also reported in Tsofliou et al.’s study, where days of attendance were significantly associated with greater intake of many nutrients, when compared with days they did not attend (all *p* values ≤ .031; Tsofliou et al., 2020). Other authors reported significant positive associations in the intake of nutrients and in overall diet ratings, dietary diversity, and dietary variety between individuals who attended the shared meals and those who did not (Choi et al., 2021; Kohrs et al., 1980; Neyman et al., 1998). Participant gender was found to mediate some of these correlations; some benefits were only relevant to female or male attendees (Huffman et al., 2017; Kohrs et al., 1980; Neyman et al., 1996, 1998).

Contrary to these positive results, many studies reported shared meal attendees did not achieve their recommended dietary intake of many nutrients, and there were many instances where no significant associations were found between groups for vitamin and mineral intake (Neyman et al., 1996), intake from some of the food groups (Choi et al., 2021) or contribution of major food groups to energy intake (Neyman et al., 1996, 1998). Additionally, Neyman, Zidenberg-Cherr et al. reported no significant association of nutrient intakes on days participants attended the meal program, compared to days they did not attend (Neyman et al., 1996). These findings were echoed by Heurberger and Wong, who reported no correlation between congregate meal attendance and nutritional intake for their population of widows (Heuberger & Wong, 2014). Similarly, Shultz et al. reported no significant association of changes to food measures between or within their regular meal program and no meal program groups (Schultz et al., 2021). Shultz et al. even found significant associations between higher healthy eating self-efficacy and frequency of vegetable intake in the no meal program group (*p* = .042, *p* = .047) compared to their innovative Encore Café program group (Schultz et al., 2021). These results indicate that the shared meal programs may positively contribute to dietary intake and meal patterns in some instances, for some population groups. However, these findings are not consistent, are based largely on correlational evidence, and it is hard to discern clear patterns.

#### Nutrition status

Five of the included papers measured outcomes related to nutrition status, or nutrition risk (Keller, 2006; Kohrs et al., 1980; Neyman et al., 1996; Schultz et al., 2021; Vailas et al., 1998). Three of these papers investigated the OAA Title III NSP (Kohrs et al., 1980; Neyman et al., 1996; Vailas et al., 1998), one explored various community care services in Canada, including congregate meal programs (Keller, 2006), and the other explored the so-called “innovative Encore Café” program compared with non-use and use of traditional congregate meal programs in the United States (Schultz et al., 2021). Kohrs et al. reported correlations between higher prevalence of less than acceptable concentrations of both vitamins A and C (*p* < .001) for those who did not attend the meal program frequently when compared with those who did (Kohrs et al., 1980). However, both Neyman, Zidenberg-Cherr et al. and Kohrs et al. reported no other significant associations between regularity or frequency of attendance and most biochemical markers (Kohrs et al., 1980; Neyman et al., 1996). Or, where differences were found, there were no observable trends between those who did or did not attend. Both Vailas et al. and Keller et al. reported associations of greater nutritional risk for those not involved in the meal program (Keller, 2006), or involved in home delivery meal programs (Keller, 2006; Vailas et al., 1998) than those who were regularly involved, with Vailas et al. reporting a significant difference between the two (*p* < .05). This sits in contrast to Schultz et al.’s study, where the majority of the participants in both the traditional congregate meal program and the innovative Encore Café program were classified as “at nutritional risk” (Schultz et al., 2021).

#### Physical health

Six studies measured outcomes related to physical health for those who attended shared meal programs (ACL, 2004, 2008, 2009, 2011–2019; Beasley et al., 2018; Choi et al., 2021; Kohrs et al., 1980; Neyman et al., 1996). Again, all but two study populations were from the OAA Title III NSP. Authors who measured physical health outcomes between attendees and non-attendees of the shared meal program reported no significant associations between appetite rating (Neyman et al., 1996), health rating (Choi et al., 2021; Neyman et al., 1996; Schultz et al., 2021), body mass index (Choi et al., 2021; Neyman et al., 1996), chewing difficulty (Choi et al., 2021), and all other anthropometrical measures (Kohrs et al., 1980). Kohrs et al. reported a significant association between attendance and prevalence of thinness in their sample of women (*p* < .05), and that a larger percentage of non-attendees were treated for obesity, gall bladder disease, heart disease, and arthritis than their frequent attendee counterparts (*p* < .05; Kohrs et al., 1980). Participants in Schultz et al.’s study reported a correlation between higher total health impact scores and weekly attendance at a meal program, regardless of whether it was their innovative Encore Café program or the regular congregate meals program (*p* = .33; Schultz et al., 2021). Seventy-four percent of participants in the 2004 ACL survey reported they were able to maintain their weight due to participation in the program, and between 64% and 75% across all the surveys reported the program improved their health (ACL, 2004, 2008, 2009, 2011–2019), which is echoed in Beasley et al.’s report of their analysis of the National ACL survey in 2015 (Beasley et al., 2018).

#### Social support and networks

Eight of the included studies measured outcomes related to social support and networks (ACL, 2004, 2008, 2009, 2011–2019; Dichiera et al., 2002; Neyman et al., 1996; Porter et al., 2016; Thomas & Emond, 2017; Tsofliou et al., 2020; Van Zandt & Fox, 1986; Ye et al., 2017). Five studies investigated the OAA Title III NSP (ACL, 2004, 2008, 2009, 2011–2019; Dichiera et al., 2002; Neyman et al., 1996; Porter et al., 2016; Van Zandt & Fox, 1986), and the remaining three explored various other shared meal programs in the United Kingdom (Thomas & Emond, 2017; Tsofliou et al., 2020) and Shanghai (Ye et al., 2017). Six of these studies measured social support and network outcomes using quantitative methods, and the remaining two adopted qualitative methods.

Participants across these studies identified making new friendships, renewing old friendships, and socializing with others as either a motivator to attend or an outcome of attending their respective shared meal programs (ACL, 2004, 2008, 2009, 2011–2019; Dichiera et al., 2002; Neyman et al., 1996; Tsofliou et al., 2020; Van Zandt & Fox, 1986). Participants in Sheppard et al.’s study described the program as a family and a community (Sheppard et al., 2018), and participants in Tsofliou et al.’s study rated socialization at the meal program higher than affordability or other activities offered (Tsofliou et al., 2020). Due to limited transport options, participants in Thomas and Emond’s study reported the shared meal program as one of the few places they could go to eat a meal out of the house (Thomas & Emond, 2017). Additionally, Ye et al.’s Shanghai study reported comradery at the shared meals, with participants enjoying their tablemates’ companionship, disclosing difficulties with them, and receiving support from them (Ye et al., 2017). Porter et al. reported a difference in experiences between their heterosexual and lesbian, gay, bisexual, transgender participants, with heterosexual participants placing higher value on the access to a social network the meals provided (*p* < .01; Porter et al., 2016). These authors also reported that those who scored lower on their loneliness scores placed a higher value on the social connection of the shared meal program (*p* < .01; Porter et al., 2016).

#### Well-being and quality of life

Seven of the included papers measured well-being and quality of life outcomes using quantitative methods (ACL, 2004, 2008, 2009, 2011–2019; Beasley et al., 2018; Huffman et al., 2017; Schultz et al., 2021; Vailas et al., 1998; Van Zandt & Fox, 1986; Ye et al., 2017). Five of these investigated the OAA Title III NSP (ACL, 2004, 2008, 2009, 2011–2019; Beasley et al., 2018; Huffman et al., 2017; Vailas et al., 1998; Van Zandt & Fox, 1986), one investigated their innovative Encore Café program compared to traditional congregate meal programs and non-attendance (Schultz et al., 2021), and one investigated shared meal programs in Shanghai (Ye et al., 2017).

Van Zandt and Fox reported that 79% of participants felt a sense of well-being from attending the shared meal program in the OAA Title III NSP (Van Zandt & Fox, 1986). Between 59% and 78% of ACL participants reported the program helped them remain living independently, which corresponds to over half of the national sample explored in Beasley’s et al.’s study reporting the shared meal program allowed them to remain living at home, as it likely comes from the same data set (ACL, 2004, 2008, 2009, 2011–2019; Beasley et al., 2018). This is echoed in Huffman et al.’s study, with 66% of participants reporting the shared meal program helped them maintain their independence, particularly those with food insecurity (*p* = .04) and those who reported consuming a minimum of half of their daily calories from the meal (*p* = .002), although this was not the case for those reporting very good or excellent health (*p* = .006) (Huffman et al., 2017).

The ACL surveys also reported 91% of participants stating the meals were something to look forward to, and that between 76% and 83% of participants felt better due to participation in the program (ACL, 2004, 2008, 2009, 2011–2019). Vailas et al. reported correlations between attending the meal program and improved quality of life (*p* < .01), quality of health (*p* < .05), depression (*p* < .05), and functional status (*p* < .001) scores, when compared with those who received home-delivered meals (Vailas et al., 1998). Participants in Ye et al.’s study in Shanghai reported relatively high life satisfaction, with companionship of others at their table (*p* < .001), disclosure to others at their table (*p* < .011), and support from others at their table (*p* < .001) all positively associated with participants’ life satisfaction (Ye et al., 2017). While total and social loneliness were not reported to change for participants in either the innovative Encore Café or comparison groups in Schultz et al.’s study, emotional loneliness improved across the 6 months of the program for participants in the innovative Encore Café group (*p* = .018), with no change noted for those who did not attend a shared meal program (Schultz et al., 2021).

## Discussion and Implications

This review set out to examine the existing literature about the widespread impacts of structured commensal eating events on adults aged 60 years and older within the community. The review deliberately sought evidence from a range of jurisdictions and aimed to capture a wide variety of structured shared meal programs. However, there is an overrepresentation of studies from the United States, and all but one of these studies out of the United States investigated the OAA Title III NSP congregate meal programs. There was also a disproportionate investigation into the impact on dietary intake and meal patterns, and less exploration into other potential benefits of engaging in shared meal programs. Nevertheless, the sample provides important information about the outcomes of several different structured shared eating programs for participants across five different countries.

Older adults are at increased risk of experiencing inadequate nutrition, largely due to reduced dietary intake as a consequence of changes to physiology and circumstance (Ahmed & Haboubi, 2010; Donini et al., 2003; Whitelock & Ensaff, 2018). Adequate nutrition is critical for maintaining health, muscle mass, and cognition for older individuals (Drewnowski & Shultz, 2001; Keller, 2004; Klimova et al., 2020; MacIntosh et al., 2000; Payette & Shatenstein, 2005). Shared meal programs are well placed to improve dietary intake and nutritional outcomes in this population. Fourteen of the 18 studies included in this review measured the impact of their shared meal program on the dietary intake and meal patterns of participants. As anticipated, almost all reported a positive effect. For many participants across these studies, the shared meal did not displace usual eating events, but rather added to overall food intake, thus increasing overall nutrient intake. However, when measured against individuals who did not attend regular shared meal programs, or who attended alternative programs, significant differences between dietary intake and meal patterns were not always observed. Additionally, some improvements were only reported or found to be significant for either males or females. This indicates that while many of these programs contributed to improved meal patterns and nutritional intake for participants, benefits were not universal, and not always clinically significant or meaningful.

Beyond the impacts on dietary intake and meal patterns, there were minimal significant associations found between shared meal programs and physical health measures, including nutritional status. This could be a result of the extra food eaten at the shared meal event not being of sufficient quality to impact on these outcomes. Alternatively, the extra meal provided at the shared meal program may not be substantial enough to impact an individual’s overall physical health or nutritional status. These findings indicate that while some participants may receive benefits to dietary intake and meal patterns from attending shared meal programs, benefits do not necessarily translate to improved physical measures of health or nutrition status. However, that is not to say that the shared meal programs do not positively impact other areas of health.

As previously described, commensal eating occasions have been shown to benefit health and well-being beyond the nutrients provided at the meal (Dunbar, 2017; Jönsson et al., 2021; Ochs & Shohet, 2006). As loneliness and social isolation are key risk factors for ill-health in older adults, shared meal programs are well situated to provide opportunities for social connection and support. The impact on social supports and networks of shared meal programs was acknowledged by several studies included in the review. Included studies reported participants making new friendships, socializing with others, creating community, disclosing difficulties, and receiving support from their tablemates as benefits from the shared meal programs. In some instances, these programs offered individuals a rare opportunity to leave the house and to eat a meal away from home (Thomas & Emond, 2017). Older adults generally have reduced participation in social activities as a result of illness, mobility issues, low energy levels, difficulties managing symptoms, and difficulties using transport (Goll et al., 2015). This reduced social participation contributes to experiences of social isolation and loneliness in older adults (Goll et al., 2015). Therefore, the opportunity for making and maintaining social connections with others provided at these shared meal programs may play a crucial role in reducing older individuals’ feelings of loneliness and social isolation. This is particularly significant when considering the evidence that the act of sharing a meal with others increases the social connections and bonds that are formed (Dunbar, 2017). As such, the benefit of the shared meal programs may not lie in the nutrition provided, but rather in their ability to combat social isolation and loneliness and their associated comorbidities.

The psychosocial outcomes of attending shared meal programs are also noteworthy. Where it was evaluated, in all instances, the impact of these shared meal programs on well-being and quality of life was positive. Although only measured by seven of the 18 studies, the evidence was consistent across different jurisdictions. Participants consistently reported better quality of life and quality of health, higher functional status, and lower levels of depression and emotional loneliness as a result of attending the shared meal programs. The shared meal programs were in many cases described by participants as improving their sense of well-being, and many perceived that the programs allowed them to remain living independently. The mental health and well-being of individuals are identified as increasingly instrumental to the health of individuals and populations (Grenade & Boldy, 2008; Nicholson, 2012). While the data pertaining to the impact on physical health and nutrition status may not have been conclusive, the studies overwhelmingly indicated that shared meals have a positive impact on self-reported quality of life and well-being for many participants and may thereby constitute a promising health promotion intervention.

With aging individuals at increased risk of experiencing deterioration to their physical and mental health, it is important that programs exist in the community that support individuals to age in place (Bigonnesse & Chaudhury, 2020; Chiu et al., 2020; Golinowska et al., 2016). This review confirms that structured shared meal programs show promise in supporting the health and well-being of older adults in the community. They provide additional nutrition, opportunities for social connection and support, and are perceived to contribute to the quality of life and perceived well-being. However, the mechanism of how they support health and well-being has not yet been identified. More work is required to understand how these shared meal programs work to facilitate health and well-being, and how they can best be used in the community to improve health outcomes for older populations.

### Strengths and Limitations

This review is strengthened by the extensive, comprehensive searching that was undertaken to identify studies. Ongoing search alerts ensured that no newly published eligible studies would be missed, and hand-searching of reference lists and gray literature searching ensured relevant studies were captured. Two reviewers checked the abstracts independently and full-text screening was conducted via a collaborative process to minimize selection bias. However, this review is not without its limitations. The majority of the included studies were cross-sectional in design, limiting the ability to perform a meta-analysis, or to understand the causal pathways and relationships between shared meal programs and the reported outcomes. As it is a scoping review, no evaluation of quality of individual studies was undertaken; however, it should be noted that many of these studies did not appear to adhere to reporting guidelines, and overall study quality was difficult to determine in many instances. To be included in this review study populations had to have a majority of participants aged over 60 years, resulting in the exclusion of gray literature reports on shared meal programs that targeted older adults, but also included participants of varying ages. Only studies published in English were included in this review, which may have resulted in the exclusion of relevant studies published in other languages. Finally, as a scoping study, many different study designs were included, and we were unable to draw any combined statistical conclusions about the findings. We were therefore unable to make judgments about the strength of the evidence of the relationship between the shared meal programs investigated and any of the health outcomes measured.

## Conclusion

Our review was undertaken to identify the scope of relevant literature in this field, explore the impact of structured commensal eating events on older individuals in the community, and examine the associated health or well-being outcomes. The nutritional and dietary benefits of commensal eating events on older individuals are well represented in the literature; however, other beneficial outcomes were measured to a lesser extent. This review has identified that future research on the social benefits of community commensal eating events is warranted to fully understand how food nurtures not only the body but also supports individuals as they age to maintain community connections, friendships, and enhance life satisfaction. With this understanding, we will be able to improve current programs, and design future programs that will effectively, and sustainably enhance the health and well-being of older adults.

## Supplementary Material

igac068_suppl_Supplementary_Materials
