# Supplementary material for: The Health and Well-being Impacts of Community Shared Meal Programs for Older Populations: A Scoping Review
Source: Innov Aging. 2022 Oct 27;6(7):igac068. doi: 10.1093/geroni/igac068 (PMC9795837; doi:10.1093/geroni/igac068)
Supplement: igac068_suppl_Supplementary_Materials [file igac068_suppl_supplementary_materials.docx]

# Online Supplementary Material

**Supplementary Table 1.** **Full electronic search strategy for Ovid MEDLINE.**

Database(s): Ovid MEDLINE(R) and Epub Ahead of Print, In-Process & Other Non-Indexed Citations, Daily and Versions(R)
Search Strategy:

| **#** | **Searches** | **Results** |
| --- | --- | --- |
| 1 | exp *Meals/ | 3117 |
| 2 | exp elder nutritional physiological phenomena/ | 224 |
| 3 | ((social or group or structur* or formal or commensal or communit* or communal or congregate or shar*) adj1 (meal* or food* or eating or dining)).tw,kf. | 4087 |
| 4 | or/1-3 | 7324 |
| 5 | aged/ or "aged, 80 and over"/ or frail elderly/ | 3098030 |
| 6 | (elder* or geriatric* or gerontolog* or old age* or grandparent* or retire* or pensioner* or senior*).tw,kf. | 389730 |
| 7 | ((old* or age* or aging) adj1 (person or people* or adult* or resident* or population* or m?n or wom?n or male* or female*)).tw,kf. | 764813 |
| 8 | (aged adj1 ("65" or "70" or "75" or "80" or "85")).tw,kf. | 41331 |
| 9 | or/5-8 | 3739296 |
| 10 | 4 and 9 | 1706 |
| 11 | (note or editorial or letter or comment or news).pt. | 2035462 |
| 12 | 10 not 11 | 1688 |
| 13 | limit 12 to english language | 1639 |

**Supplementary Table 2. Detailed findings of the included studies.**

| **STUDY** | **REPORTED IMPACT ON HEALTH OR WELL-BEING** |
| --- | --- |
| Kohrs 1980; USA | **DIETARY INTAKE / MEAL PATTERNS**  Frequency of attendance sig. assoc. (*p*<0.05) with increased levels of dietary intake of riboflavin and thiamine  Frequency of attendance sig. assoc. (*p*<0.05) with increased intake of Vitamin A and ascorbic acid rich fruit and vegetables  Frequency of attendance sig. assoc. (*p*<0.05) with increased ingestion of vitamin A rich fruit and vegetables for women  Frequency of attendance sig. assoc. (*p*<0.05) with higher likelihood of reaching breads and cereals intake recommendations for men  Overall diet rating was positively and sig. assoc. (*p*<0.05) with attendance in the program  **NUTRITION STATUS**  Higher prevalence of less than acceptable concentrations of Vitamins A (*p*<0.001) and C (*p*<0.001) for those who did not attend frequently  No sig. assoc. between frequency of attendance and other biochemical markers  **PHYSICAL HEALTH**  Prevalence of thinness sig. assoc. (*p*<0.05) with attendance for women  No sig. assoc. between attendance and any other anthropometrical measures  Larger percentage of non-attendees treated for obesity, gall bladder disease, heart disease and arthritis (*p*<0.05) |
| Van Zandt 1986; USA | **DIETARY INTAKE / MEAL PATTERNS** 40% had a change in meal patterns since attending 91% reported program provided good food  Felt that meals provided nutritional balance  Assumed food provided was sufficient for the day, or the meal + home diet would be sufficient  Many stated the program helped control food costs  **SOCIAL SUPPORT / NETWORK**  87% stated they met new friends at the lunches  68% reported renewing old friendships  **WELL-BEING / QUALITY OF LIFE**  79% felt a sense of well-being from participating |
| Neyman 1996; USA | **DIETARY INTAKE / MEAL PATTERNS**  More non-attendees (87%) ate three meals a day than did attendees (72%)  Intake of energy for female attendees was above the recommended dietary allowance  No sig. difference between groups for protein or dietary fat contribution of energy intake  Carbohydrates provided greater proportion of energy for female attendees compared with male attendees and male non-attendees (*p*<0.05)  Greater intake of dietary cholesterol for male attendees and non-attendees than female attendees and non-attendees (*p*<0.05)  No sig. difference between groups for vitamin and mineral intake  No sig. difference in nutrient intakes on days at meal centre vs. days not at meal centre  **NUTRITION STATUS**  Nutritional status within normal ranges for all groups, except iron higher than normal across all groups  Several sig. differences in nutrition status were noted with respect to attendees vs non-attendees and/or men vs women, but no general trends observed  **PHYSICAL HEALTH** No difference between how attendees and non-attendees rated their appetites or health No sig. difference between body mass index values for attendees and non-attendees  **SOCIAL SUPPORT / NETWORK**  Att. consistently cited “to socialise with others” and “to eat a meal” as the 1^st^ and 2^nd^ reasons why they attended |
| Neyman 1998; USA | **DIETARY INTAKE / MEAL PATTERNS** Male attendees ate more total energy and carbohydrate than non-attendees (*p*<0.05) No sig. difference in %age of total energy contributed by macronutrients between groups  Mean intake of dietary cholesterol all groups less than recommendation  Mean fibre intake less than recommendation, except for male attendees  Male attendees consumed more Vitamins A, C and E, folate, iron, and magnesium than non-attendees (*p*<0.05)  Mean intakes of Vitamin B6 <2/3 recommended dietary allowance (RDA) for male non-attendees  Vitamin C consumed at <RDA by male non-attendees  Vitamin E consumed at <RDA by attendees and at <2/3 by non-attendees  Calcium consumed at <RDA by female attendees and all non-attendees  Magnesium consumed <RDA by female attendees and non-attendees and <2/3 by male non-attendees  Zinc consumed at <RDA by attendees and <2/3 by non-attendees More non-attendees than attendees consumed several nutrients at <2/3 RDA, with a sig. difference for folate (*p*<0.05) |
| Vailas 1998; USA | NUTRITION STATUSGreater nutritional risk for HDM than CMS (*p*<0.05) **WELL-BEING / QUALITY OF LIFE**  HDM less well-off regarding quality of life (*p*<0.01), quality of health (*p*<0.05), depression (*p*<0.05), and functional status (*p*<.001) than CMS  Spearman correlation coefficient for quality-of-life scores and CMS participation *r*=-.45 (*p*<0.0001), and HDM participation *r*=-.29 (*p*<0.05) indicating larger impact for CMS participation |
| Dichieria 2002; USA | **DIETARY INTAKE / MEAL PATTERNS**  56% reported nutritional quality of meals consumed at the centre better than at home, 44% reported it was the same, and 1% reported it was worse  63% reported enjoying the meals as a reason for attending the lunches  57% reported inexpensive meals as a reason for attending the lunches  88% who ate at the centre at least 3 times per week described the nutritional quality of the meal as better than a meal they would consume at home (*p*=.003)  Participants who selected “nutritional quality” as a reason for coming to the lunches, compared with those who did not, were more likely to consume vegetables at the lunches (*p*=.03)  When eating at home, participants’ intakes were lower in fruits, vegetables, and dairy foods, and higher in fats/sweets and meat  **SOCIAL SUPPORT / NETWORK**  61% reported enjoying socialisation as a reason for attending the lunches |
| Administration for Community Living 2003, 2004, 2008, 2009, 2011-2019; USA | **DIETARY INTAKE / MEAL PATTERNS**  74-75% reported they ate more balanced meals at the program  70-80% reported they ate healthier meals at the program  55% reported the program made it easier to keep to special diets  76% reported they were better able to avoid sodium/fat at the program  **PHYSICAL HEALTH**  74% reported they were able to maintain their weight because of participation in the program  64-75% reported the program improved their health  **SOCIAL SUPPORT / NETWORK**  92% reported enjoying mealtime more because of the company of others  81-87% reported seeing their friends more as a result of the program  **WELL-BEING / QUALITY OF LIFE**  91% reported the meals as something to look forward to  59-78% reported participation in the program helped them stay living independently 76-83% report feeling better because of participation in the program |
| Keller 2006; Canada | **NUTRITION STATUS**  Greater percentage not involved in meal program at high nutritional risk at follow-up (42.3%), followed by CMS (33%), and MOW (29.7%)  Those who participated in MOW or CMS had higher nutritional risk scores at follow-up than nonparticipants had at baseline (only MOW participation was sig. (*p*=0.04)) |
| Heuberger 2014; USA | **DIETARY INTAKE / MEAL PATTERNS**  No sig. association found between congregate meals and nutrition intake |
| Porter 2016; USA | **DIETARY INTAKE / MEAL PATTERNS**  All participants rated the nutritional value of the lunch highly  HS participants placed higher value of the provision of the nutritional meal itself relative to LGBT participants (*p*<.001) – when controlling for income the assoc. is no longer sig.  **SOCIAL SUPPORT / NETWORK**  All participants rated communal value of the lunches and access to network value of the lunches highly  HS participants placed higher value on access to the network provided via CMS (*p*<0.01)  Those reporting less loneliness placed higher value on social connection of CMS (*p*≤0.01)  HS participants placed a higher value on having access to a social network compared with LGBT attendees when controlling for loneliness, partner status, age, gender, and income |
| Thomas 2016; United Kingdom | **SOCIAL SUPPORT / NETWORK**  Lunch club one of the few places participants could go in order to eat out due to limited mobility and transport options  No participant stated that quality of the meal was a motivating or valued aspect of attending the lunch club  Sociability of the lunch club was prioritised, and gratified, over and above the material content of meals |
| Huffman 2017; USA | **DIETARY INTAKE / MEAL PATTERNS**  80% thought the meals helped them to eat healthier  Majority said they eat vegetables at the CMS  Higher proportion of males had >50% daily calories coming from CMS and considered they ate healthier because of them  Fewer participants who reported having very good/excellent health, thought they were eating healthier because of the meals (*p*=0.001)  **WELL-BEING / QUALITY OF LIFE**  66% thought the meals helped them with their independence  Odds of reporting that meals helped maintain independence were higher for those with food insecurity (*p*=0.040) and those who reported eating at least half of their calories from the site (*p*=0.002)  Fewer participants who reported having very good/excellent health, thought the meals helped with their independence (*p*=0.006) |
| Ye 2017; China | **SOCIAL SUPPORT / NETWORK**  Participants had average 2 tablemates, enjoyed tablemates’ companionship, disclosed difficulties to tablemates and received support from tablemates  **WELL-BEING / QUALITY OF LIFE**  Participants portrayed relatively high life satisfaction  Number of tablemates had no sig. assoc. with participants’ life satisfaction  After adjusting for individual-level factors, tablemates’ companionship (*p*<.001), disclosure to tablemates (*p*<.011), and instrumental support from tablemates (*p*<.001) were assoc. with participants’ life satisfaction |
| Beasley 2018; USA | **DIETARY INTAKE / MEAL PATTERNS**  **National Sample**  77.5% stated meals improved their diet  81.5% stated meals helped them feel better  **NYC Sample**  32% met total fat target  14% met saturated fat target  14% met protein target  14% met cholesterol target  22% met calcium target  22% met magnesium target  31% met sodium target  28% met dietary fibre target  **PHYSICAL HEALTH**  **National Sample**  74.4% reported meals improved their health  **WELL-BEING / QUALITY OF LIFE**  **National Sample**  58.1% stated meals help them to remain living at home |
| Sheppard 2018; Canada | **DIETARY INTAKE / MEAL PATTERNS**  Participants described the program as an opportunity to enjoy healthy, tasty, inexpensive, and convenient meals  **SOCIAL SUPPORT / NETWORK**  Participants noted the program felt like a family and/or community  Participants indicated sharing a meal with others was prioritised above the food served (with some offering to bring their own food to eat if they did not like what was offered at the venue) |
| Tsofliou 2020; United Kingdom | **DIETARY INTAKE / MEAL PATTERNS**  Important factors relating to lunch experience: hot meal (74.4%), dining outside the home (76.9%), home-style cooked meal (71.8%), skipping cooking (43.6%)  Lunch days were sig. assoc. with greater intake of energy, protein, fat, carbohydrate, saturated fatty acids, fibre, potassium, calcium, iron, Vitamins A and C, folate, and water from drinks (all *p* values ≤0.031)  **SOCIAL SUPPORT / NETWORK**  Important factors relating to lunch experience: meeting with friends (92.3%)  Meal affordability and the activities at the lunch not deemed important factors |
| Choi 2021; South Korea | **DIETARY INTAKE / MEAL PATTERNS**  Higher intake of seafood, seaweeds, dairy products, grains and eggs in IFS group than non-IFS group (all *p*≤.048)  No sig. difference between groups for other food groups  Higher water intake in IF group than non-IF group (*p*<.001)  Higher intake of protein, fat, dietary fibre, calcium, iron, phosphorus, potassium, vitamin A, thiamine, riboflavin, niacin, and sodium in IF group than non-IF group (all *p*≤.024)  Higher carbohydrate contribution to total energy in non-IF group than IF group (*p*<.001)  Higher protein and lipid contribution to total energy in IF group than non-IF group (all *p*≤.002)  No sig. difference between groups for nutrient adequacy ratio (NAR) of energy or vitamin C  Higher NAR of protein, calcium, iron, phosphorus, vitamin A, thiamine, riboflavin and niacin in IF group than non-IF group (all *p*≤.024)  Higher dietary diversity scores and dietary variety scores in IF group than non-IF group (*p*<.001)  Higher grain + meat + veg pattern in IF group than non-IF group  **PHYSICAL HEALTH**  Mean body mass index 18.9kg/m2 for all participants, no sig. difference between groups  48.4% of all participants rated themselves to be in ‘moderate’ health with no sig. difference between groups  39.1% of all participants reported finding chewing to be ‘uncomfortable’ but no sig. difference between groups |
| Schultz 2021; USA | **DIETARY INTAKE / MEAL PATTERNS**  No sig. changes from pre to post (6 months) or any between group differences for any food measures detected for Encore Café or comparison group  Healthy eating self-efficacy was sig. higher in comparison group than Encore Café group at post (*p*=0.042)  Comparison group had sig. increase in frequency of vegetable intake from pre to post (6 months) (*p*=0.047)  Encore Café group had lower intake frequencies of processed meats compared to comparison group at post (*p*=0.043), however no change in intake from pre to post (6 months)  **NUTRITION STATUS**  Majority of participants in comparison and Encore Café group were classified as “at nutritional risk” pre and post (6 months)  **PHYSICAL HEALTH**  High health impact scores reported at pre and post (12 months) for both Encore Café and traditional groups  Total health impact score at post (12 months) for all respondents regardless of Encore café or traditional group positively correlated with weekly attendance at a lunch program (*p*=0.033)  No sig. changes from pre to post (6 months) or any between group differences for health measures detected for Encore Café or comparison group  **WELL-BEING / QUALITY OF LIFE**  Total and social loneliness did not change for either Encore Café or comparison group from pre to post (6 months)  Emotional loneliness improved from pre to post (6 months) for Encore Café group (*p*=0.018) when controlling for housing type, with no change noted for comparison group |
